# The Role of Cyclo(His-Pro) in Neurodegeneration

**DOI:** 10.3390/ijms17081332

**Published:** 2016-08-12

**Authors:** Silvia Grottelli, Ilaria Ferrari, Grazia Pietrini, Matthew J. Peirce, Alba Minelli, Ilaria Bellezza

**Affiliations:** 1 Dipartimento di Medicina Sperimentale, Università di Perugia, Polo Unico Sant’Andrea delle Fratte, Piazzale Gambuli, 06132 Perugia, Italy; mattpeirce69@gmail.com (M.J.P.); alba.minelli@unipg.it (A.M.); ilaria.bellezza@unipg.it (I.B.); 2 Dipartimento di Biotecnologie Mediche e Medicina Traslazionale, Università degli Studi di Milano ed Istituto di Neuroscienze, Consiglio Nazionale delle Ricerche, Via Vanvitelli 32, 20129 Milano, Italy; Ilaria.ferrari87@hotmail.it (I.F.); grazia.pietrini@unimi.it (G.P.)

**Keywords:** oxidative stress, endoplasmic reticulum stress, neuroinflammation

## Abstract

Neurodegenerative diseases may have distinct genetic etiologies and pathological manifestations, yet share common cellular mechanisms underpinning neuronal damage and dysfunction. These cellular mechanisms include excitotoxicity, calcium dysregulation, oxidative damage, ER stress and neuroinflammation. Recent data have identified a dual role in these events for glial cells, such as microglia and astrocytes, which are able both to induce and to protect against damage induced by diverse stresses. Cyclo(His-Pro), a cyclic dipeptide derived from the hydrolytic removal of the amino-terminal pyroglutamic acid residue of the hypothalamic thyrotropin-releasing hormone, may be important in regulating the nature of the glial cell contribution. Cyclo(His-Pro) is ubiquitous in the central nervous system and is a key substrate of organic cation transporters, which are strongly linked to neuroprotection. The cyclic dipeptide can also cross the brain-blood-barrier and, once in the brain, can affect diverse inflammatory and stress responses by modifying the Nrf2-NF-κB signaling axis. For these reasons, cyclo(His-Pro) has striking potential for therapeutic application by both parenteral and oral administration routes and may represent an important new tool in counteracting neuroinflammation-based degenerative pathologies. In this review, we discuss the chemistry and biology of cyclo(His-Pro), how it may interact with the biological mechanisms driving neurodegenerative disease, such as amyotrophic lateral sclerosis, and thereby act to preserve or restore neuronal function.

## 1. Introduction

Neurodegenerative diseases such as Alzheimer’s disease, amyotrophic lateral sclerosis (ALS), Huntington’s disease (HD) and Parkinson’s disease (PD), are late-onset multifactorial disorders with a progressive loss of function of neurons, which leads to a progressive functional decline. More than 30 million people world-wide are affected, most commonly in their seventh decade, while in the EU, the proportion of the population aged 65 or over is predicted to increase from 15.4% to 22.4% by 2025. Currently available therapies provide only symptomatic relief and completely fail to address the likely inflammatory basis of these diseases. The academic and pharmaceutical research is currently focused on the discovery of novel drugs for the treatment of neurodegenerative disorders.

The past decades have seen a growing awareness of the role of peptide transmitters and regulators in biological systems [1,2,3,4,5,6,7,8,9,10,11,12]. More recently, one particular class, cyclic dipeptides (CDPs), or 2,5-diketopiperazines, have been the subject of intense research interest. CDPs are simple compounds derived from the non-enzymatic cyclisation of dipeptides and their amides. Thyrotropin-releasing hormone (TRH), a tripeptide synthesized by the hypothalamus, acts as a neuroendocrine signal that elicits many behavioral responses [13]. In addition, accumulating data now point to a significant neuroprotective role of TRH. For example, substantial literature [1,14,15,16] reports that traumatic brain or spinal cord injuries can be significantly improved by TRH or TRH analogues. The catabolic product of TRH is the (His-Pro) dipeptide, which, by spontaneous cyclization, produces cyclo(His-Pro), and is the topic of this review. Here, we focus on the potential protective role of cyclic dipeptide (His-Pro) in neurodegeneration, beginning with an overview of the biochemistry and biology of cyclo(His-Pro), followed by an overview of the diverse biological mechanisms of neurodegeneration and, finally, describing how cyclo(His-Pro) may modulate these mechanisms to protect, or even restore, neuronal function.

## 2. Cyclo(His-Pro): Chemistry and Biology

**Figure 1:**
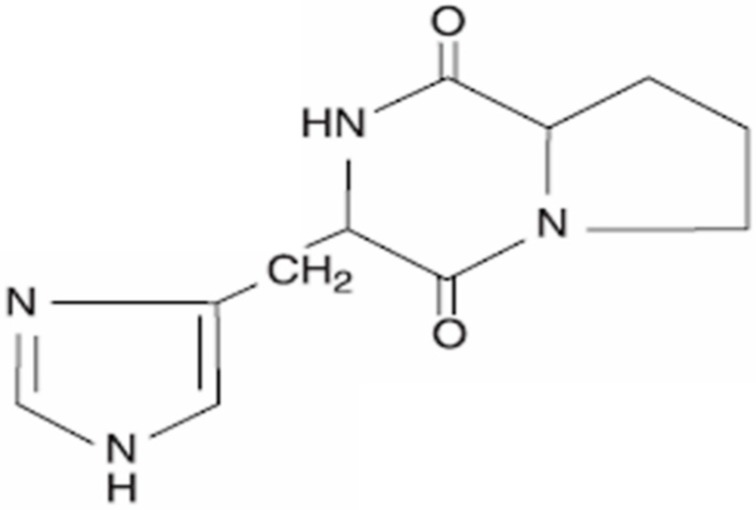
Cyclo(His-Pro) chemical structure.

The major mechanism responsible for the extracellular inactivation of TRH within the CNS is the hydrolytic removal by pyroglutamyl aminopeptidases (PPs) of the amino-terminal pyroglutamic acid residue [17]. Following cleavage, the His-Pro-NH_2_ dipeptide undergoes cyclization at 37 °C by a non-enzymatic pH-dependent process (optimal at pH 6.0 to 7.0) [18], producing the cyclic dipeptide histidyl-proline (cyclo(His-Pro)). This cyclization reaction confers resistance to cleavage by peptidases and is also required for its active transport in the intestine [19] and the passage of the blood brain barrier (BBB) [20,21,22], a key characteristic for delivery and specific targeting of cyclo(His-Pro) therapy in the CNS. Adding to their suitability as therapeutic agents, cyclic dipeptides often exhibit significantly greater stability than their linear counterparts in vivo. While cyclo(His-Pro) can be generated from TRH as described above, the large majority is in fact endogenously synthesized de novo. In addition, cyclo(His-Pro) also possesses its own unique receptors, metabolic pathways and biological effects [23]. Cyclo(His-Pro) (Figure 1) is ubiquitous in the CNS and has been found in blood, in the gastrointestinal (GI) tract, as well as in several body fluids [24]. As first reported by Perry and colleagues [18], cyclo(His-Pro)-like immunoreactivity (CHP-LI) has been identified in foods and in several common nutritional supplements [25], while dietary intake of CHP-LI-rich supplements in healthy volunteers were reported to increase the levels of cyclo(His-Pro) in plasma significantly above the baseline values (1848 ± 117 pg/mL vs. 2148 ± 112 pg/mL) [26]. Importantly for the potential therapeutic application of these agents, acute consumption of 24 mg cyclo(His-Pro)/die can be absorbed from the GI tract without any toxicity in humans weighing an average of 70 kg [27].

### Cyclo(His-Pro) as a Key Substrate of Organic Cation Transporter

The mechanisms of the reabsorption and excretion of drugs were studied after discovering mammalian drug efflux transporters of the ATP binding cassette (ABC) family [28,29], such as the H^+^/oligopeptide cotransporter family SLC15 [30], the organic anion transporting family SLC01 [31,32], the organic cation/anion/zwitterion transporter family SLC22 [33] and the multidrug and toxin extrusion (MATE) H^+^/drug antiporters [34]. Polyspecific organic cation transporters belong to the SLC22 family and the MATE family [35]. The SLC22 family comprises three subtypes of passive diffusion organic cation transporters, called OCT1 (SLC22A1), OCT2 (SLC22A2) and OCT3 (SLC22A3), characterized by 12 α-helical transmembrane domains, an intracellular N-terminus, two extracellular loops and an intracellular C-terminus [36]. OCTs translocate a variety of organic cations in either direction [37]. Cyclo(His-Pro) has structural features essential for OCT transport. Indeed, cyclo(His-Pro) is selectively transported by OCT2 in the brain [36]. OCT2 is mostly expressed in the dopaminergic brain regions, particularly in substantia nigra pars compacta (SNc). It is perhaps worth noting that this is also the brain area associated with one of the commonest neurodegenerative diseases, Parkinson’s disease. With the exception of kidney, where OCT2 may be involved in the clearance of cyclo(His-Pro), OCT2 levels in peripheral tissues are all considerably lower than in SNc [36,38,39]. The distribution of cyclo(His-Pro) itself in rat brain reveals striking coincidence with both dopaminergic areas and OCT2 consistent with the potential functional link previously reported in the literature [23,40]. Structure/function studies have identified the proline residue and the presence of unsaturated systems as structural elements contributing to the nootropic and cognitive-enhancing properties, as well as the overall neuroprotective action of the natural/synthetic cyclic dipeptides [2,3,4,5,6,11,41,42]. Pretreatment of OCT2-transfected HEK-293 cells, SH-SY5Y and HTZ-146 cells with cyclo(His-Pro) prior to neuronal insult substantially diminished cell degeneration, by inhibiting excitotoxic calcium influx and its damaging sequelae: mitochondrial impairment and subsequently apoptosis [37]. Therefore, high expression of OCT2, as well as that of cyclo(His-Pro) are crucial for the maintenance of dopaminergic cell integrity. A decline in protective cyclo(His-Pro) may underpin calcium-triggered apoptotic cell death, possibly contributing to the selective chronic nigral degeneration observed in Parkinson’s disease [37].

## 3. Role of Cyclo(His-Pro) in Common Mechanisms of Neurodegeneration

### 3.1. Oxidative and Nitrosative Stress

Neurons are extremely susceptible to oxidative stress because of their terminally-differentiated state and complex morphology. They depend largely on surrounding glial cells for metabolic substrates and glutathione [43,44]. Thus, the brain is highly sensitive to changes in redox status, and maintaining redox homeostasis is critical for preventing oxidative damage. Because of the limited capacity of the neurons to protect themselves, the bulk of this critical task falls to the glial cells. In the absence of the redox homeostasis provided by glial cells, oxidative stress (OS) and nitrosative stress (NS) thus result in the accumulation of oxidized molecules and the disruption of normal neuronal processes. Energy generation via the process of oxidative phosphorylation in the mitochondrial electron transport chain is the main endogenous source of reactive oxygen species (ROS). Indeed, mitochondrial dysfunction, strongly associated with neurodegenerative diseases, leads to increased ROS generation while decreasing ATP production. Moreover, in the context of failing mitochondria, NADPH oxidase yields superoxide anions, which combined with nitric oxide in this setting produced largely by inducible nitric oxide synthase (iNOS), generates the highly reactive peroxynitrite (RNS) [45,46,47,48]. The activity of iNOS is largely controlled by its transcription [49], which requires the activation of the nuclear factor-κB (NF-κB) that, in turn, may be influenced by ROS [50,51,52]. NF-κB is composed of p65 and p50 heterodimers, which are maintained in an inactive form in the cytosol by association with the IκB family. The stimulation of cells with pro-inflammatory agents causes the phosphorylation of IκBα, resulting in its polyubiquitination and proteasomal degradation [53]. Thus released from IκBα, p65/p50 heterodimers are able to enter the nucleus, driving the expression of cell adhesion molecules and pro-inflammatory factors [54]. Moreover, NF-κB is a redox-sensitive transcription factor that drives the expression of genes governing inflammation, growth and apoptosis [50,51,55]. Oxidative/nitrosative damage can affect nucleic acids, proteins and lipids. Markers of OS and NS are a defining feature of all neurodegenerative diseases, strongly suggesting a causal link between ROS/RNS and neurodegeneration [47,56,57,58,59]. To counteract these stresses, the cells must maintain their cellular redox homeostasis via a complex interplay of many redox-sensitive transcription factors, which together orchestrate the expression of an array of protective genes [50,51,60]. The pathway of the Nrf2 (nuclear factor erythroid 2-related factor 2) antioxidant response element (ARE) is critical for this protective response. Nrf2 is sequestered in the cytosol by Keap1 (Kelch ECH-associating protein), an actin-bound protein [61,62]. Keap1, a Cul3-based E3 ligase, polyubiquitinates Nrf2, triggering its proteasomal degradation [63,64]. Upon oxidative stress, Keap1 cysteine residues are modified, thus releasing Nrf2 that translocates to the nucleus, binds ARE sequences and upregulates several antioxidant genes [65,66].

NF-κB and Nrf2-signalling pathways are activated by several physiological and/or pathological stimuli. On the other hand, anti-inflammatory and/or anti-carcinogenetic compounds suppress NF-κB and activate the Nrf2 signaling pathways [67,68,69,70,71,72]. ROS levels are critical determinants of cell fate. Indeed, chronically-elevated ROS levels induce cell death, activate NF-κB and lead to inflammation [73,74], whereas moderate ROS levels activate Nrf2 and lead to the upregulation of stress-inducible genes, such as heme oxygenase-1 (HO-1) [10]. HO activity exerts anti-inflammatory and adaptive survival responses upon oxidative insults [10,71,75,76,77,78] suggesting that anti-inflammatory and anti-oxidant pathways are coordinated through a complex mechanism. We showed that cyclo(His-Pro) protected dopaminergic PC12 cells from oxidative stress by activating the Nrf2-ARE pathway. Indeed, cyclo(His-Pro) augmented the expression of several ARE-containing genes. Moreover, cyclo(His-Pro) reduced ROS production and prevented glutathione depletion induced by rotenone, paraquat and β-amyloid treatment, suggesting that the dipeptide may act as an antioxidant compound [9,10]. Consistent with this possibility, we also showed that cyclo(His-Pro) abolished hydrogen peroxide-mediated ROS and NO generation and glutathione depletion, which lead to apoptotic cell death [10]. The mechanism of protection against cellular redox stress is due to both the thioredoxin system, which regulates the redox status of protein thiols involved in signal transduction and gene regulation, and the glutathione system, which maintains a low redox potential and high free thiol levels [79]. Cyclo(His-Pro) upregulates genes related to both redox systems (glutathione-synthesizing/regenerating enzymes and thioredoxin-1 isoform), thus indicating that cyclo(His-Pro) acts as an Nrf2-inducing agent. It is to note that the increase in mitochondrial ROS generation due to the disturbance of glutathione metabolism is implicated in both ageing and neurodegenerative disorders [44,80]. Cyclo(His-Pro) counteracted the hydrogen peroxide-mediated increase in NO production (along with the expression of NOS isoforms), while at the same time preventing glutathione depletion, suggesting that cyclo(His-Pro) may be a potential therapeutic agent in oxidative stress-based diseases [9].

Recently, the antioxidant properties of cyclo(His-Pro) were studied in microglial cells overexpressing the mutated human gene SOD1G93A, which are used as a glial model of ALS [81]. By exposing microglial SOD1G93A cells to an oxidative stressor such as paraquat, we found that the exogenous oxidative stress worsens the neurotoxic effect of the mutated microglia cells, confirming the contribution of ROS to disease progression. More importantly, we observed that the use of cyclo(His-Pro), by reducing the oxidative burden and triggering the protective response, was able to partially attenuate ROS toxicity.

### 3.2. Endoplasmic Reticulum Stress

In the endoplasmic reticulum (ER), proteins are folded multi-subunit protein complexes, lipids are assembled, sterols are synthesized and calcium is stored. Various stressful environmental stimuli including, calcium dysregulation and OS, can alter ER function leading to the accumulation of unfolded/misfolded proteins within the lumen of the ER. These events trigger the unfolded protein response (UPR) [82]. The UPR is regulated by ER-resident proteins, i.e., inositol-requiring enzyme 1 (IRE1), PKR-like endoplasmic reticulum kinase (PERK) and activating transcription factor (ATF) 6. The downstream activation of all three pathways is important both in protective or adaptive responses to protein accumulation, but also in the promotion of apoptosis through the expression of various apoptotic activators, such as C/EBP-homologous protein (CHOP). The decision to induce an adaptive or pro-apoptotic response depends on the accumulation of misfolded proteins and the duration of the stress exposure [83]. Short-term stress and moderate misfolded protein accumulation induce the UPR, whereby accumulated misfolded proteins are cleared either through the ER-associated degradation (ERAD) machinery linked to the ubiquitin proteasome system (UPS) or through autophagy, restoring cellular homeostasis [83]. Longer term stress and/or severe protein accumulation might result in cell death rather than adaptive cell maintenance programs. For example, neurodegenerative diseases have been linked to the constitutive activity of the ER stress response [83,84,85,86,87,88,89,90,91,92]. Our results showed that cyclo(His-Pro) attenuates ER stress in BV-2 microglial cells [69]. The inhibitor of protein glycosylation, tunicamycin, failed to induce detectable NO, but it led to a concentration-dependent decrease in cell viability, which was reduced by treatment with cyclo(His-Pro). This effect was linked to the cyclo(His-Pro)-mediated early activation of three UPR transducers, thus increasing the phosphorylation of the α subunit of eIF2α, responsible for initiating the UPR. Furthermore, it increased the protein levels of Bip (GRP78), an ER chaperone, while decreasing the levels of the apoptosis-inducer CHOP. Whereas tunicamycin caused a biphasic response in NF-κB nuclear translocation, cyclo(His-Pro) treatment induced a stable NF-κB nuclear translocation. On the other hand, nuclear translocation of Nrf2, a known PERK substrate [93], was enhanced by cyclo(His-Pro). These results show that cyclo(His-Pro) can modulate the Nrf2–NF-κB axis when present at the same time as an ER stressor.

LPS is known as an inducer of ER stress [69,94,95]. Treatment with cyclo(His-Pro) hastened the LPS-induced activation of the three UPR transducers, while at the same time increasing Bip levels and increasing the phosphorylation of eIF2α. Notably while CHOP protein levels were strongly upregulated by the ER stress-inducer tunicamycin, they were undetectable following treatment with LPS with or without cyclo(His-Pro), demonstrating qualitative differences in the cellular responses to these two different ER stressors [95]. These results indicate that cyclo(His-Pro) increases the sensor of ER stress and launches the UPR designed to alleviate the ER stress by upregulating Bip.

### 3.3. Excitotoxicity and Calcium Overload

Neuronal excitotoxicity like other cellular responses to major stresses can ultimately lead to neuronal death, which is a conserved pathological feature of neurodegenerative diseases, including Huntington’s disease (HD), Alzheimer’s disease (AD), Parkinson’s disease (PD) and amyotrophic lateral sclerosis (ALS) [96,97,98,99,100,101]. Excitotoxicity stems from the excessive release of the neurotransmitter glutamate, which in turn causes post synaptic over excitation of neurons. One of the mechanisms involved is calcium overload, a result of excessive glutamate signaling, which, by activating calcium-dependent enzymes and increasing ROS and RNS, results in cell death [102,103]. In our Lab, we observed that dopaminergic PC12 cells, exposed to a high concentration of glutamate/hydrogen peroxide, showed robust increases in intracellular calcium levels, which, along with increases in ROS and NO generation and decreases in glutathione levels, eventually resulted in cell death. These changes were significantly reversed by pre-treatment with cyclo(His-Pro), thus increasing cell survival [9], and demonstrate the protective effect of cyclic dipeptide against glutamate toxicity.

## 4. Neuroinflammation

The term neuroinflammation defines a situation characterized by a broad range of immune responses within the CNS, driven primarily by cross-talk between microglia, astrocytes and the BBB. The BBB is an active player in neuroinflammation since it responds to peripheral inflammatory stimuli, generates inflammation mediators and allows leukocyte migration. Thus, peripheral inflammation might be one of the causes of damaging neuroinflammation. The neuroinflammatory response impairs synaptic transmission and causes neuronal death. Microglial cells play a crucial role in the process of neuroinflammation. Indeed, in response to mediators of acute inflammation, microglia, the resident macrophages of the CNS, assume an amoeboid phagocytic state, with abundant filopodia, and release additional pro-inflammatory mediators. Long-lasting microglial activation is toxic to neighboring neurons, and the resulting neuronal damage can itself further amplify microglial activation and initiate a self-propelling cycle of inflammation and progressive neuronal damage [104]. Astrocytes, the other class of glial cell, respond to CNS insult through reactive astrogliosis, characterized by progressive changes in gene expression and other cellular changes [105,106,107,108,109,110]. Excessive and prolonged neuroinflammation abolishes the capacity of astrocytes to maintain brain function and, thus, is relevant to CNS disease progression. In conclusion, neuroinflammation can initiate, amplify and prevent the normal resolution of acute stress responses, promoting the chronic conditions that result in neurodegeneration.

### 4.1. Cyclo(His-Pro) Acts as an Anti-Inflammatory Agent

Dopaminergic PC12 cells, exposed to strong oxidative stressor, responded to the insult by increasing the nuclear translocation of both Nrf2 and NF-κB transcription factors. Pre-treatment with cyclo(His-Pro) increased the nuclear level of Nrf2 and, by blocking IκB-α degradation, inhibited NF-κB nuclear translocation, promoting adaptive responses while reducing potentially damaging apoptotic and inflammatory responses and confirming the interplay between the suppression of NF-κB signaling and the activation of the Nrf2 pathway [111]. Notably, HO-1 induction has been shown to be protective in various experimental models of vascular, cardiac and pulmonary injury, as well as against damage from certain inflammatory conditions [75,76,77,78]. Cyclo(His-Pro), via Nrf2 activation, increased HO-1 expression, elevated HO activity and protected PC12 cells from ROS toxicity [10], thus suggesting that HO activity resulting in increased production of antioxidant end-products suppresses ROS-mediated NF-κB activation. Indeed, conclusive evidence of the correlation between the anti-oxidant and anti-inflammatory effects of cyclo(His-Pro) was obtained with in vivo experiments by subjecting mice to a model of acute skin edema induced by a single topical application of TPA (12-*O*-tetradecanoylphorbol 13-acetate), to the ear. Cyclo(His-Pro) pre-treatment significantly inhibited the TPA-induced edema response confirming its anti-inflammatory effects.

### 4.2. Cyclo(His-Pro) Reduces Microgliosis/Neuroinflammation

**Figure 2:**
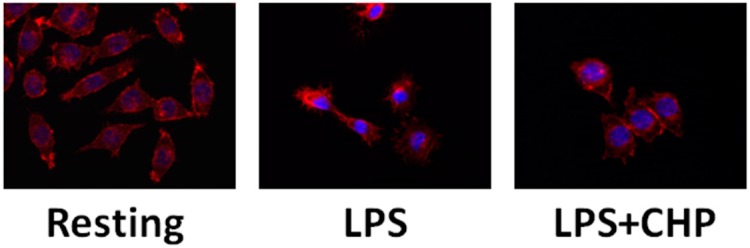
Effects of cyclo(His-Pro) (CHP) on glia morphology. BV2 microglial cells were pre-treated with 50 μM cyclo(His-Pro) (24 h) prior to 10 µg/mL lipopolysaccarde (LPS) (24 h). Morphology was assessed by TRIC-labelled phalloidin staining (red) and nuclei were counterstained with DAPI (blue). Magnification: 20×.

In LPS-treated microglial BV-2 cells [69], pre-treatment with cyclo(His-Pro) was able to reduce LPS-induced NO and ROS generation while increasing cell viability and largely maintaining resting morphology (Figure 2).

**Figure 3:**
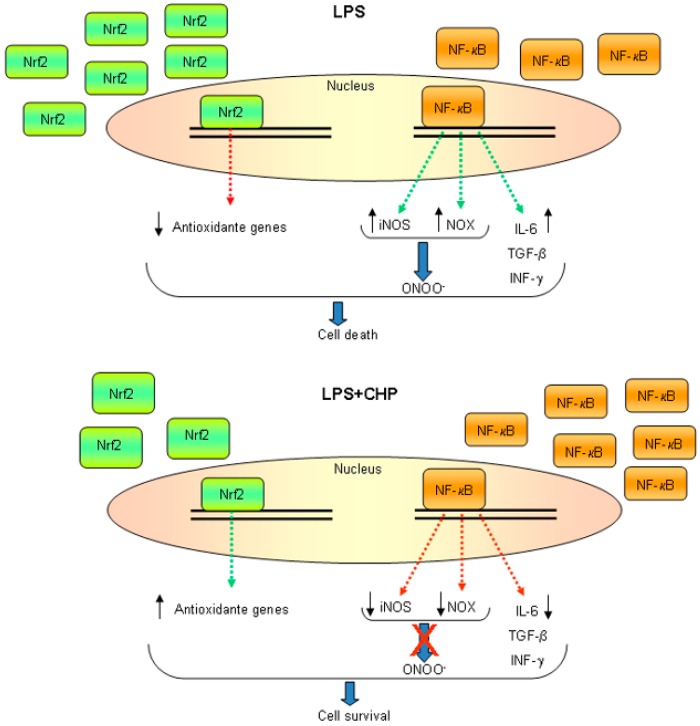
Proposed glia nuclear events in the presence of cyclo(His-Pro). NF-κB (nuclear factor kappa B), NOX (NADPH oxidase), Nrf2 (nuclear factor erythroid 2–related factor 2), iNOS (inducible nitric oxide synthase), IL-6 (interleukin 6), ONOO (peroxynitrite), TGF-β (transforming growth factor beta), INF-γ (interferon gamma). Red X indicates inhibition of peroxynitrite production by cyclo(His-Pro). ↑ indicate an increase; ↓ indicate a decrease.

Moreover, the increased nuclear localization of NF-κB coupled with the reduced nuclear localization of Nrf2, which normally follow LPS treatment of these cells, were at least partially reversed by cyclo(His-Pro); levels of nuclear Nrf2 were enhanced while those of NF-κB were reduced. These data confirm the ability of cyclo(His-Pro) simultaneously to activate endogenous antioxidant defenses and inhibit pro-inflammatory pathways. At the transcriptional level, these changes in transcription factor nuclear localization were reflected in strongly downregulated inflammatory gene expression (iNOS and COX-2 (cyclooxygenase 2)) and augmented protective responses (HO-1 expression). It is to note that cyclo(His-Pro) also reduced the expression of the key enzymes in superoxide generation, the NADPH oxidase membrane-bound subunit (gp91phox) and the NADPH oxidase organizer subunit (47phox) (Figure 3). These results are in agreement with the dual-key mechanism of inflammatory neurodegeneration [112]. In addition to these effects reducing the capacity to induce oxidative cell damage, we also found [69] that cyclo(His-Pro) reduced the inflammatory capacity of these cells; the production of several pro-inflammatory cytokines, such as IL-6, TGF-β and INF-γ, was reduced and, moreover, protected primary neuronal cultures against microglial neurotoxicity, i.e., pro-inflammatory/neurotoxic factors contained in LPS-activated BV-2 culture media. These results were largely recapitulated in vivo by showing that cyclo(His-Pro) also reduced glial inflammation caused by systemic LPS administration. Analysis of the animals by Fourier transform infrared (FTIR) spectroscopy showed that those treated with cyclo(His-Pro) before LPS exposure were strikingly similar to the control animals and were markedly different from LPS-treated animals. Moreover, in vivo cyclo(His-Pro) treatment strongly reduced mRNA levels of TNFα in liver and brain, as well as mRNA levels of Il-1β in hippocampus. Collectively, these results provided strong evidence for the in vivo glial anti-inflammatory properties of the cyclic dipeptide [69].

## 5. Role of Cyclo(His-Pro) in in Vitro Models of Familial Amyotrophic Lateral Sclerosis

Amyotrophic lateral sclerosis (ALS) is a devastating neurodegenerative disorder that results in progressive loss of motor function and ultimately death. Ten percent of ALS cases are inherited, while the rest are considered sporadic. Twenty percent of inherited ALS is caused by mutations in the gene encoding for superoxide dismutase 1 (SOD1) providing further evidence of the connection between neurodegeneration and oxidative stress. While it is now clear that all of the different mutations of SOD1 associated with pathology augment rather than inhibit its function, the molecular mechanisms leading to motor neuron damage remain to be defined [113]. A role for non-neuronal cells, such as microglia and astrocytes, has been suggested for ALS pathogenesis by experimental and clinical observations, thus opening a new area for identifying potential therapeutic targets [114,115].

**Figure 4:**
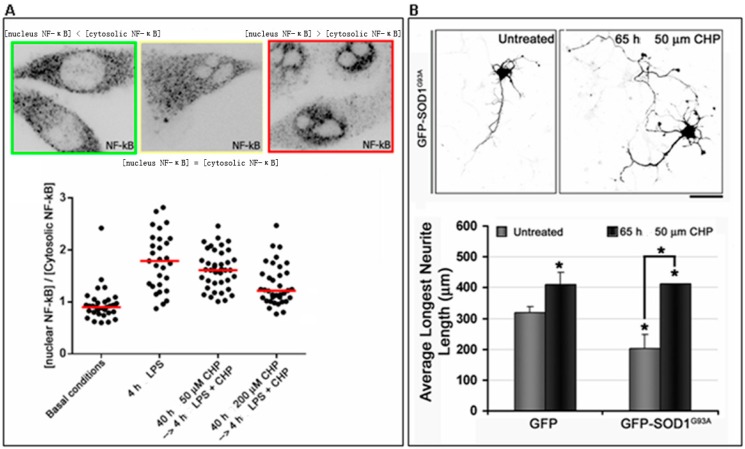
Role of cyclo(His-Pro) in ALS. (**A**) Cyclo(His-Pro) prevents the toxic effects of mutant SOD1 in microglia. Microglial cells immortalized from neuronal primary cultures derived from SOD1^G93A^ transgenic mice were pre-treated with 50 or 200 μM cyclo(His-Pro) (40 h) prior to 1 µg/mL LPS exposure in serum-free medium. Representative immunofluorescence images were quantitatively analyzed to classify the cells into three different categories on the basis of the NF-κB distribution between the nucleus and cytoplasm (magnification: 65×). The scatter plot of the effects of increasing the concentration of cyclo(His-Pro) on NF-κB distribution; (**B**) Cyclo(His-Pro) prevents the toxic effects of mutant SOD1 in neurons. Primary cortical neurons were transfected after one day in vitro with GFP or GFP-SOD1^G93A^ and fixed with paraformaldehyde at Day 4 in vitro. Representative immunofluorescence images of neurons expressing GFP-SOD1^G93A^ untreated or treated for 65 h with 50 μm cyclo(His-Pro) are shown. Bar: 50 μm. Images of GFP- or GFP-SOD1^G93A^ transfected neurons were quantitatively analyzed to assess the length of the longest neurite. Data represent the mean ± s.e.m. of at least three independent experiments. Student’s *t*: * *p* < 0.05 vs. GFP-transfected neurons.

Given our findings on the anti-inflammatory and anti-oxidative effects of cyclo(His-Pro), we tested whether cyclo(His-Pro) might reduce the capacity of microglial cells to induce inflammatory and/or oxidative neuronal damage and thereby protect against neurodegeneration in ALS [116]. First, by using microglial cell lines from cortical cultures derived from human SOD1^G93A^ transgenic mice, immortalized in our laboratory, we observed increased levels of nuclear NF-κB in cells expressing the G93A mutant of human SOD1 compared to wild-type control lines. In line with microglial activation induced by the mutant SOD1, we measured a high nuclear/cytoplasmic ratio of NF-κB in the immortalized microglia from SOD1^G93A^ cultures, even under basal conditions (Figure 4A). Upon LPS challenge, the nuclear/cytoplasmic ratio doubled, whereas in the presence of 50 μM cyclo(His-Pro), this ratio was reduced, and 200 μM cyclo(His-Pro) completely prevented the LPS-induced NF-κB nuclear translocation (Figure 4A). These data further demonstrate the in vitro anti-inflammatory properties of cyclo(His-Pro) and suggest that these properties may be effective against inflammatory responses resulting from SOD1 mutants associated with ALS.

To investigate the potential neuroprotective mechanism of cyclo(His-Pro), primary cultures of rat cortical neurons (kindly provided by Drs. Verpelli C. and Sala C., Consiglio Nazionale delle Ricerche (CNR)-Institute of Neuroscience of Milano) were transfected with cDNA encoding human SOD1^G93A^. In these cells, we observed that striking defects in neurite outgrowth, when compared to mock transfected GFP cells, were largely inhibited in the same cells cultured in medium supplemented with 50 μM cyclo(His-Pro) (Figure 4B).

Collectively, these data indicate that cyclo(His-Pro) may inhibit the neuronal damage associated with SOD1 mutations both indirectly, at the level of microglial inflammatory responses, and by direct effects on neurons themselves, suggesting its possible utility as a therapeutic agent to prevent or delay disease progression in ALS. Pre-clinical trials are needed to confirm the promising in vitro data.

## 6. Conclusions

Cyclo(His-Pro) is a dipeptide with much greater stability in vivo than its linear counterpart and thus shows much greater promise as a therapeutic agent. Conventional anti-inflammatory therapeutics are mostly unsuitable for treating neuroinflammation since they cannot cross the BBB. Being a BBB-permeable drug, cyclo(His-Pro) can be administered by both parenteral and oral routes, thus increasing patients’ compliance. The dipeptide shows a remarkable bioactivity in reducing inflammatory responses in glial cells and in inducing a protective state in neurons. As discussed here, published data provide a dual-pronged justification for the therapeutic use of cyclo(His-Pro) against neuroinflammation-related diseases.
